# Data on emerging sulfur dioxide in the emission of natural gas heater in winter

**DOI:** 10.1016/j.dib.2018.09.030

**Published:** 2018-09-15

**Authors:** Reza Fouladi Fard, Amir Hossein Mahvi, Mohsen Mahdinia, Monireh Dehabadi

**Affiliations:** a Research Center for Environmental Pollutants, Qom University of Medical Sciences, Qom, Iran; b School of Public Health, Tehran University of Medical Sciences, Tehran, Iran; c National Institute of Health Research, Tehran University of Medical Sciences, Tehran, Iran; d Center for Solid Waste Research, Institute for Environmental Research, Tehran University of Medical Sciences, Tehran, Iran

**Keywords:** Natural gas heater, Air pollution, SO_2_, Cold days

## Abstract

Natural gas is a kind of fuel that is used in various heating systems for combustion processes. Combustion of natural gas produce such air pollutants as CO_2_, NOx, SOx, PM, CO, and HC. During cold days, total gas consumption in Iran goes up. Thus, in these days it is likely to make some changes in gas properties that can affect the emissions from gas heaters. Portable flue gas analyzer (LANCOM III) was used for, SO_2_, NO_X_, and C_X_H_Y_ measurement from December 2012 to April 2013. Data show that emissions of pollutants from the gas heater were different during the cold seasons. On colder days, total gas consumption in the country increased, SO_2_ emerged in heater stack, and the concentration of hydrocarbons (C_X_H_Y_) significantly increased as well. This emergence could be attributed to the changes in gas properties in the colder days. In these days, the transient flow and high changes of speed and pressure in the gas pipes could lead to release of some deposited sulfur in gas flow. Therefore, sulfur dioxide will be generated in the combustion process.

**Specifications table**

**Table:** 

Subject area	Environmental Science
More specific subject area	Air pollutant emission of a natural gas heater
Type of data	Table, text file, and figure
How data was acquired	Gaseous pollutant measurement was done by portable flue gas analyzer (LANCOM III)
Data format	Raw and analyzed.
Experimental factors	Sampling site are located at least eight stack diameters downstream and two diameters upstream. the flow of inlet gas to the heater was fixed on 10 l/min. Measurement was done twenty minutes after heater ignition
Experimental features	Measurement of pollutants (SO_2_, C_X_H_Y_, and NOx) was done two or three days a week and three times in each day with LANCOM III. Daily ambient temperature and total daily network gas consumption data were obtained from Qom Meteorological Office and National Iranian Gas Company (NIGC), respectively
Data source location	Qom province, Iran.
Data accessibility	The data are available within this article.
Related research article	Ko and Lin [1]

**Value of the data**

• Considering this research, sulfur dioxide emerges in the exhaust of gas heaters during among cold days.
• The data will be useful for finding the contribution of household sources to urban air pollution.
• The data of this research can be used by gas companies and the Ministry of Petroleum.
• The data obtained from this research can be used by the Environmental Protection Agency and the Ministry of Health and Medical Education.
• The data will be useful for researchers in fields of combustion, fuel, and air pollution.

## 1 Data

**Fig. 1:**
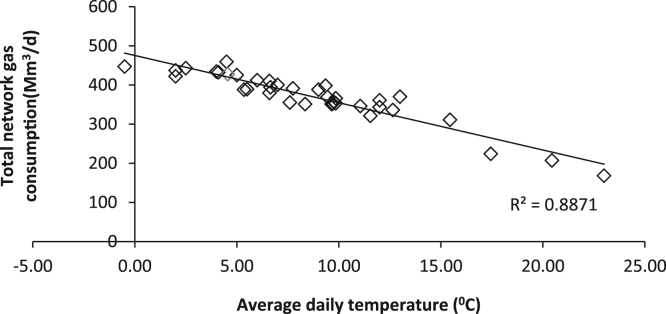
The relation between average daily temperatures with total daily network gas consumptions.

The presence of pollutants in breathing air could be as a matter of industrial activities, work places or domestic areas [2], [3], [4], [5]. The Household gas heater is one of the most widely used domestic heating systems in Iran. Although household heaters are individually small in size, they can contribute significantly to production of greenhouse gases (GHG). Because of the large number of these devices, particularly in developing countries, the household use is a significant portion of the total fuel consumption [6]. Combustion of natural gas produce such air pollutants as CO_2_, NOx, SOx, PM, CO, and HC [7]. These pollutants are the main air pollution problems in big cities [8], [9]. The estimates show that in some very cold winter days, the level of total network gas consumption is more than 1.5 times as much as the daily average of warm and temperate months [10]. Due to fluctuations in gas consumption from difference in electricity generation, seasonal variations in consumption, short-term gas sales contracts, etc., it is not possible to analyze the gas network flow with a steady state circumstance [11], [12]. Clogging and sedimentation of sulfur when the pressure and temperature are reduced in gas pipelines, well head facilities, dehydration unit, and gas sweetening unit are plausible [11]. During the cold days, when total gas consumption in the country has gone up, transient flow condition of the gas in the pipes occurs and changes of speed and pressure in the pipes increase. Therefore, in these days it is likely to make some changes (according to the deposition of sulfur in the gas pipes and possibly less purified gas) in gas properties that can affect the emissions of from gas heaters. The data of SO_2_ (micro g/m^3^_)_, NOx (micro g/m^3^), and C_X_H_Y_ (ppm) in natural gas heater exhaust with total network gas consumption (Mm^3^) and average daily temperature (°C) are presented in Table 1. Fig. 1 demonstrates the relation between average daily temperatures data with total daily network gas consumptions. According to this figure, when the average daily temperature decreases, total daily gas consumption through the country arises. Pearson analysis shows that there is a highly significant reverse relation between these variables. (*p*-value < 0.0001). Table 2 shows the Pearson correlation statistics between gas heater exhaust pollutants (SO_2_, C_X_H_Y_, and NOx) with total network gas consumption and average daily temperature data. There are significant negative relations between SO_2_ and C_X_H_Y_ with average daily temperature and total network gas consumption. On the days when the weather was too cold, total gas consumption in the country increased, SO_2_ emerged in heater stack (*p*-value = 0.002), and the concentration of hydrocarbons (C_X_H_Y_) significantly increased (*p*-value = 0.001). This emergence could be due to the changes in gas properties in the colder days. In these days, transient flow and high changes of speed and pressure in the gas pipes lead to release of some deposited sulfur in gas flow [12], [13], [14]. Although there are rumors that in the cold days, there is a shortage of sweet gas in the country, some less purified gas (containing sulfur or sour gas) are added to the network for compensating this shortage.

**Table 1:** Data of daily average concentration of natural gas heater exhaust pollutants with total network gas consumption and average daily temperature.

Date of sampling	Average daily temperature (°C)	Total network gas consumption (Mm^3^)	Average concentration of SO_2_ (micro g/m^3^)	Average concentration of NOx (micro g/m^3^)	Average concentration of C_X_H_Y_ (ppm)
12/06/2012	9.70	353	0.00	9915.41	300.00
12/09/2012	9.75	357	0.00	19,257.20	433.33
12/11/2012	9.85	366	0.00	1460.86	166.67
12/13/2012	6.60	380	0.00	1072.11	100.00
12/16/2012	4.55	428	660.51	16,513.08	333.33
12/18/2012	4.10	432	0.00	12,668.31	366.67
12/23/2012	9.00	388	294.79	16,178.70	400.00
12/25/2012	5.50	389	490.57	11,762.66	333.33
12/27/2012	2.00	422	913.35	14,687.79	333.33
12/30/2012	2.00	437	913.35	14,687.79	200.00
01/01/2013	2.50	443	0.00	1310.59	333.33
01/02/2013	4.00	434	567.96	1232.95	333.10
01/06/2013	5.00	425	0.00	1481.51	300.00
01/08/2013	6.00	412	0.00	1633.73	166.67
01/10/2013	7.00	400	0.00	9713.82	433.33
01/13/2013	−0.50	447	1176.18	11,047.10	400.00
01/15/2013	4.50	459	0.00	1786.02	200.00
01/21/2013	9.35	398	0.00	2249.48	100.00
01/24/2013	12.00	361	0.00	3192.13	133.33
02/02/2013	6.65	394	0.00	9941.81	166.67
02/04/2013	6.60	410	0.00	2733.69	66.67
02/06/2013	7.75	391	0.00	1081.91	0.00
02/09/2013	12.00	343	0.00	1408.40	0.00
02/11/2013	11.55	321	0.00	14,621.13	100.00
02/13/2013	8.35	351	0.00	11,773.18	333.33
02/16/2013	9.65	351	0.00	1294.75	0.00
02/18/2013	9.85	353	0.00	1952.85	0.00
02/20/2013	11.05	346	117.12	16,996.07	166.67
02/23/2013	7.60	355	1020.79	14,248.40	0.00
02/25/2013	9.45	369	0.00	615.64	0.00
03/04/2013	15.45	311	0.00	719.45	0.00
03/06/2013	12.65	336	0.00	536.48	0.00
03/09/2013	5.35	388	0.00	563.24	0.00
03/11/2013	13.00	370	0.00	638.00	0.00
04/06/2013	17.45	224	0.00	14,833.77	166.67
04/08/2013	20.45	207	0.00	14,666.66	33.33
04/10/2013	23.00	168	0.00	13,707.57	0.00

**Table 2:** Correlation between gas heater exhaust pollutants with total network gas consumption and average daily temperature.

Exhaust pollutants	Statistics	Total network gas consumption	Average daily temperature
SO_2_	*r*	0.340*	−0.502**
	*p*	0.040	0.002
C_x_H_y_	*r*	0.469**	−0.535**
	*p*	0.030	0.001
NO_x_	*r*	−0.242	0.080
	*p*	0.149	0.638

* Correlation is significant at the 0.05 level.

** Correlation is significant at the 0.01 level.

## 2 Experimental design, materials, and methods

A conventional household gas heater in the laboratory of Health Faculty of Qom University of Medical Science selected for the emissions analyses. Sampling was performed at a site (a hole drilled for sampling with analyzer probe) located at least eight stack diameters downstream and two diameters upstream [15]. A portable flue gas analyzer (LANCOM III) was used for pollutants (NO_X_, SO_2_, and C_X_H_Y_) measurement from December 2012 to April 2013. At the sampling period, the flow of inlet gas to the combustion chamber of the heater was fixed on 10 l/min (adjusted with the control valve). Calibrated flow meter (Platon model) was used for gas flow measurement. Measurements were done two or three days a week and three times in each day, twenty minutes after heater ignition. Daily ambient temperature and total daily network gas consumption data were obtained from Qom Meteorological Office and National Iranian Gas Company (NIGC), respectively. SPSS software (Ver.22) were used for data analysis.
