# Supplementary material for: Data on emerging sulfur dioxide in the emission of natural gas heater in winter
Source: Data Brief. 2018 Sep 15;20:1764–8. doi: 10.1016/j.dib.2018.09.030 (PMC6169424; doi:10.1016/j.dib.2018.09.030)
Supplement: Supplementary file 1 — Transparency document [file mmc1.docx]

Dear Editor

There is nn conflict of interest for any of authors in the paper with regard to the manuscript.

Thanks

Mahvi
